# Abnormal visuomotor processing in schizophrenia

**DOI:** 10.1016/j.nicl.2015.08.005

**Published:** 2015-09-25

**Authors:** Siân E. Robson, Matthew J. Brookes, Emma L. Hall, Lena Palaniyappan, Jyothika Kumar, Michael Skelton, Nikolaos G. Christodoulou, Ayaz Qureshi, Fiesal Jan, Mohammad Z. Katshu, Elizabeth B. Liddle, Peter F. Liddle, Peter G. Morris

**Affiliations:** a Sir Peter Mansfield Imaging Centre, School of Physics and Astronomy, University of Nottingham, University Park, Nottingham NG7 2RD, UK; b Centre for Translational Neuroimaging in Mental Health, Institute of Mental Health, School of Medicine, University of Nottingham, Jubilee Campus, Triumph Road, Nottingham NG7 2TU, UK; c Kevin White Unit, Smithdown Health Park, Smithdown Road, Liverpool L15 2HE, UK; d Herschel Prins Centre, Glenfield Hospital, Leicestershire Partnership NHS Trust, Groby Road, Leicester LE3 9QP, UK

**Keywords:** Schizophrenia, Magnetoencephalography, Motor cortex, Visual cortex, Electrophysiological processes

## Abstract

Subtle disturbances of visual and motor function are known features of schizophrenia and can greatly impact quality of life; however, few studies investigate these abnormalities using simple visuomotor stimuli. In healthy people, electrophysiological data show that beta band oscillations in sensorimotor cortex decrease during movement execution (event-related beta desynchronisation (ERBD)), then increase above baseline for a short time after the movement (post-movement beta rebound (PMBR)); whilst in visual cortex, gamma oscillations are increased throughout stimulus presentation. In this study, we used a self-paced visuomotor paradigm and magnetoencephalography (MEG) to contrast these responses in patients with schizophrenia and control volunteers. We found significant reductions in the peak-to-peak change in amplitude from ERBD to PMBR in schizophrenia compared with controls. This effect was strongest in patients who made fewer movements, whereas beta was not modulated by movement in controls. There was no significant difference in the amplitude of visual gamma between patients and controls. These data demonstrate that clear abnormalities in basic sensorimotor processing in schizophrenia can be observed using a very simple MEG paradigm.

## Highlights

• Visual and motor deficits in schizophrenia are rarely investigated.
• We use MEG to non-invasively assess the neural basis of these deficits.
• Patients showed abnormalities in neuronal oscillations in motor cortex.
• Beta band power, reflecting cortical inhibition, was reduced after movements.
• Increased movement frequency may be a behavioural compensation for this reduction.

## 1 Introduction

Schizophrenia is a psychiatric disorder characterised by a range of symptoms including hallucinations, delusions, disorganised thought and behaviour, and reduced cognitive and emotional capacity. Research tends to focus on these core symptoms; however, patients also experience impairments in more basic sensorimotor processes (Bombin et al., 2005; Butler et al., 2001; Vrtunski et al., 1986). Abnormalities in motor function have been noted since the earliest descriptions of the disorder (Kraepelin, 1921) and are a well-accepted feature of schizophrenia, with the vast majority of patients exhibiting at least one type of motor symptom (Peralta et al., 2010; Walther et al., 2012). These symptoms include involuntary movements, catatonia, Parkinsonism and deficits in the production of both simple and complex movements such as coordination, reflexes and motor sequencing (Bombin et al., 2005; Kraepelin, 1921; Vrtunski et al., 1989). Similarly, patients with schizophrenia exhibit deficits in low-level visual function, particularly in processing stimuli of low spatial frequencies, as evidenced by reduced contrast sensitivity, centre-surround interference and abnormal motion perception (Butler et al., 2001; Cadenhead et al., 2013; Keri et al., 2002; Slaghuis, 1998).

There is significant evidence that these subtle abnormalities in basic sensorimotor processing are present in childhood, at the onset of core symptoms and in relatives of individuals with schizophrenia (Chen et al., 2000; Walther et al., 2012; Whitty et al., 2009), indicating that they are likely to be inherent to the disorder rather than being a consequence of long-term exposure to medication. Importantly, visual and motor deficits, as well as other neurological abnormalities, correlate with the primary symptoms of schizophrenia such as affective flattening, apathy and disorganisation (Bombin et al., 2005; Jahn et al., 2006; Liddle, 1987; Peralta et al., 2010), and with illness severity (Jahn et al., 2006), social functioning (Dickerson et al., 1996; Jahn et al., 2006; Lehoux, 2003) and functional outcome (Boden et al., 2014; Javitt, 2009), suggesting that they could be used as a biomarker for the disorder.

Understanding the neuronal basis of these symptoms could therefore ultimately contribute to development of treatments permitting improved quality of life; however, at present the neuronal mechanisms underlying sensorimotor processing deficits in schizophrenia are not known. It is likely that different types of symptoms have different aetiologies (Chen et al., 2000). Visual deficits have been reported to be due to abnormalities in lower-level visual pathways, particularly in magnocellular neurons (Butler et al., 2001). These neurons rely on N-methyl-d-aspartate (NMDA)-type glutamate receptors, which may show dysfunctional transmission in schizophrenia (Javitt, 2009). A review of motor symptoms and their potential aetiology by Walther and Strik (2012) describes reductions in volume of the anterior cingulate cortex and midbrain structures (putamen, caudate and thalamus), and disturbed gamma-aminobutyric acid (GABA)-ergic neurotransmission in these areas and the primary motor cortex. Neuroimaging techniques are of great use in measuring the structural and physiological abnormalities that may contribute to sensorimotor abnormalities in schizophrenia.

Magnetoencephalography (MEG) allows non-invasive inference of current flow in neuronal cell assemblies through measurement of extracranial magnetic fields. MEG signals are dominated by oscillations, which result from rhythmic activity in large populations of neurons. Neuronal oscillatory responses to visual and motor stimulation have been well characterised in healthy volunteers: in motor cortex, the amplitude of beta (13–30 Hz) oscillations decreases during movement (event-related beta desynchronisation (ERBD)) and increases above baseline on movement cessation (post-movement beta rebound (PMBR)), returning to baseline ~4 s after movement offset (Pfurtscheller et al., 1999). In the visual cortex, a decrease in alpha (8–12 Hz) oscillatory amplitude occurs alongside a concomitant increase in gamma (30–70 Hz) oscillations (Siegel et al., 2010). Notably, individual differences in the amplitude of motor beta oscillations correlate with electromyogram measures of muscle control (Jain et al., 2013; Mima et al., 2000), whilst visual gamma oscillations correlate with orientation discrimination performance (Edden et al., 2009). Measurement of these electrophysiological features is therefore likely to offer insight into the neuronal basis of motor and visual deficits in schizophrenia.

Previous studies have identified electrophysiological visuomotor abnormalities in schizophrenia and related disorders: Wilson et al. (2011) showed that adolescents with early-onset psychosis exhibit enhanced ERBD and reduced PMBR whilst conducting a motor task. Since beta oscillations are thought to reflect inhibition (Cassim et al., 2001; Gaetz et al., 2011), reduced amplitude may reflect a greater degree of processing required to plan and execute movements in patients. In visual cortex, either no change (Uhlhaas et al., 2006) or a reduction in amplitude (Grutzner et al., 2013) and frequency (Spencer et al., 2004) of gamma oscillations have been reported in schizophrenia. However, available data are sparse and typically relate to complex stimuli (e.g. faces or Gestalt stimuli) that require integration of visual features. The question of whether patients with schizophrenia show abnormalities in oscillations reflecting low-level visual and motor processing therefore remains. In this study, we measure ERBD and PMBR in sensorimotor cortex and gamma oscillations in the visual cortex during a simple visuomotor task, to test the hypothesis that these well characterised phenomena are perturbed in schizophrenia.

## 2 Methods

### 2.1 Participants

The study received ethical approval from the National Research Ethics Service and all participants gave written informed consent. The patient group was recruited from community-based mental health teams in Nottinghamshire, Derbyshire and Lincolnshire, United Kingdom. Diagnoses were made in clinical consensus meetings through a review of case files and a standardised clinical interview (Signs and Symptoms of Psychotic Illness or SSPI; Liddle, 2002) in accordance with the procedure of Leckman et al. (1982). All patients were in a stable phase of illness with no change in antipsychotic, antidepressant, or mood- stabilising medications, nor a change of more than 10 points in occupational and social function scored according to the Social and Occupational Function Assessment Scale (SOFAS) (APA, 1994), in the 6 weeks prior to the study. Patients were taking a range of psychotropic medication, with a mean defined daily dose (DDD) of 1.8 (SD 1.3) (Table 2). Controls were selected to match the patient group in terms of demographic variables. There were seventeen male and six female patients with schizophrenia and the same number of male and female controls. There was no significant difference between the ages of the two groups (patients and controls' mean ages 26.8 (SD 7.0) and 26.7 (SD 7.2), respectively; U = 264.5, p = 1.0). Groups were also matched for socio-economic background using the National Statistics Socio-economic Classification (NS-SEC) self-coded method. NS-SEC scores are given in Table 1 and did not differ significantly between groups (χ^2^(4, N = 46) = 2.3, p = .69). All participants had normal or corrected to normal vision.

### 2.2 Symptom severity measurement

In order to derive a score for overall severity of psychotic illness in the patient group, we followed the procedure employed by Palaniyappan et al. (2013a). We computed the first principal component of: the scores for the three characteristic syndromes of schizophrenia (reality distortion, psychomotor poverty and disorganisation) assessed using the SSPI; speed of cognitive processing assessed using a variant of the Digit Symbol Substitution Test (Wechsler, 1940); and scores from the Social and Occupational Function Scale (SOFAS; APA, 1994). Unlike Palaniyappan et al. (2013b), who focussed on chronic symptom burden, we did not include duration of illness in our measure of current illness severity.

### 2.3 Paradigm

The task comprised visual stimulation with a centrally-presented maximum contrast vertical square wave grating (3 cycles per degree), which subtended an 8° visual angle and was displayed behind a red fixation cross on a mean luminance background. The grating was presented for 2 s followed by a 7 s fixation only baseline period. Participants were instructed to press a button with their right index finger regularly but as many times as they chose during the 2 s presentation of the grating, though ensuring that they did not press so vigorously as to cause their arm to move. There were 45 trials, giving a total task length of 7 min. A short practice of the task was given outside the scanner. Stimuli were generated on a PC using MATLAB (The Mathworks, Inc., Natick, MA) and were back-projected via a mirror system onto a screen inside a magnetically shielded room at a viewing distance of 46 cm. All participants were scanned in a supine position. Right index finger button presses were recorded via a response pad (Lumitouch Photon Control Response System).

### 2.4 Data acquisition and analysis

MEG data were obtained using a 275 channel whole head CTF system (MISL, Coquitlam, Canada), with four channels switched off due to excessive sensor noise. Twenty-nine reference channels were also recorded for noise cancellation purposes and the primary sensors were analysed as synthetic third order gradiometer measurements (Vrba et al., 2001). Data were acquired at a csampling frequeny of 600 Hz with a 150 Hz low-pass anti-aliasing hardware filter. The position of the head within the MEG helmet was measured continuously during the recording by energising three electromagnetic head position indicator coils located at the nasion and left and right pre-auricular points, allowing continuous head movement tracking throughout the acquisition. A 3-dimensional digitisation of the head shape and fiducial locations was obtained using an Isotrak (Polhemus Inc., Vermont) system prior to the MEG measurement. All participants also underwent an anatomical MRI scan, acquired using a Philips Achieva 7 T system with a volume transmit and 32 channel receive head coil. A 1 mm isotropic image was obtained using an MPRAGE sequence (TE/TR = 3/7 ms, FA = 8°). Coregistration of the MEG sensor geometry to the anatomical MR image was achieved by fitting the digitised head surface to the equivalent head surface extracted from the anatomical MR image.

**Fig. 1:**
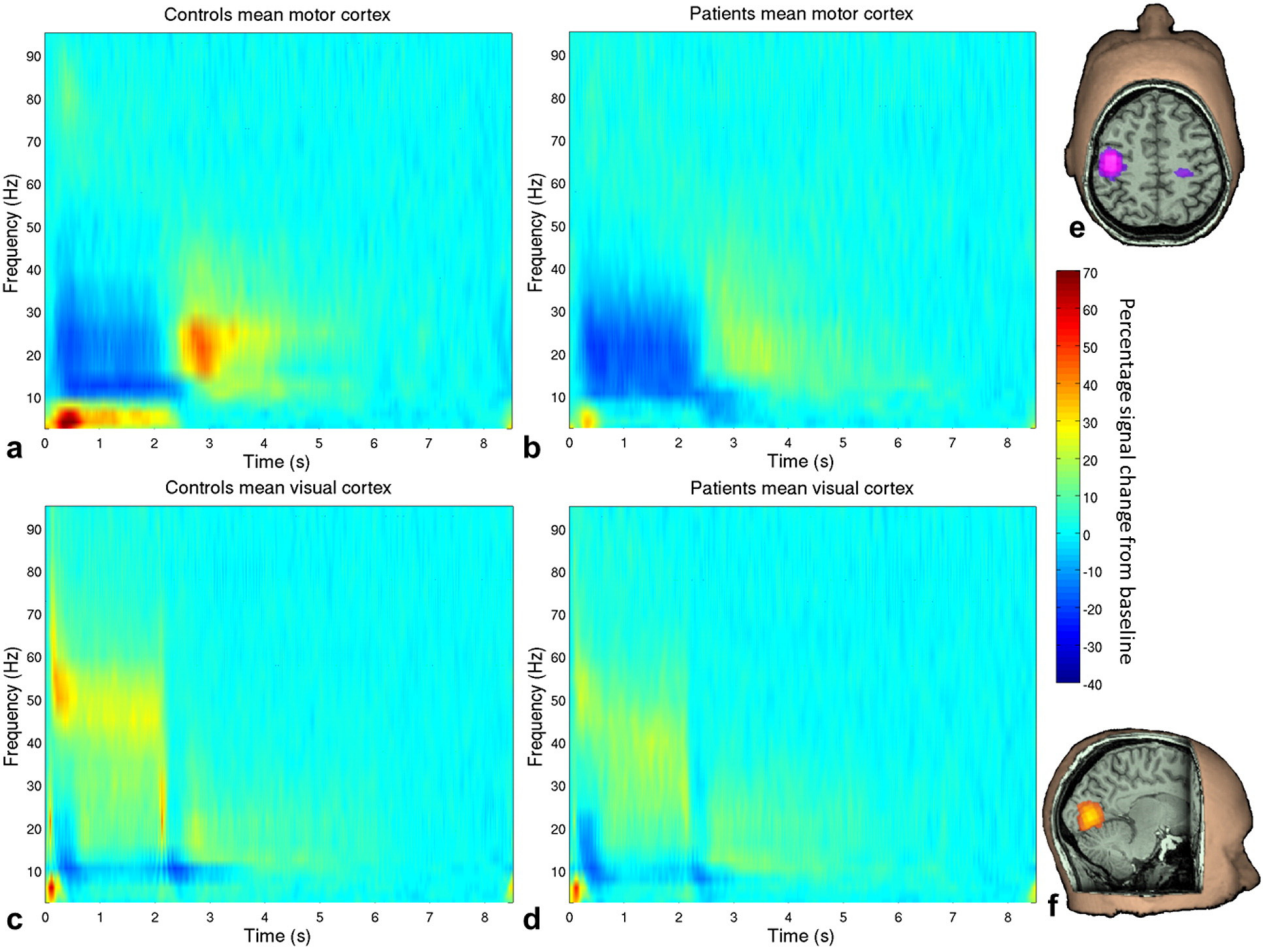
Time frequency spectrograms. Percentage change from baseline in the trial-averaged signal at the locations of individuals’ peak decrease in motor beta (a&b) and increase in visual gamma (c & d) during stimulation. Data are averaged across controls (a & c) and patients (b & d). Visual stimulation and motor responses were from 0–2 s. On the right are example pseudo t-statistical images from a single representative subject showing the spatial signature of the beam-formed signal in the stimulus window (0.5–1.8 s) contrasted with a baseline window (7–8.3 s) in the beta (13–30 Hz) band (e) and gamma (30–70 Hz) band (f).

Initially, MEG data were inspected visually. Common sources of interference, for example the magnetomyogram, magnetooculogram and magnetocardiogram, have well characterised neuromagnetic signatures which are easily identified by an experienced operator. Here, any trials deemed to contain excessive interference generated via such sources were removed from that individual's data (see also Gross et al., 2013). Head movement was assessed via continuous head localisation and any trials in which the head was found to be more than 7 mm (Euclidean distance) from the starting position were excluded. This left an average of 42 trials (SD 3.6) in controls and 38 (SD 4.9) trials in patients. Lead fields were computed individually for each participant using a multiple-local-sphere head model (Huang et al., 1999) and the dipole model derived by Sarvas (1987). A scalar beamformer (synthetic aperture magnetometry; Robinson et al., 1998) was used to project extracranial field signals into source space. Images showing the spatial signature of task induced oscillatory power change were computed in the beta (13–30 Hz) and gamma (30–70 Hz) ranges. In both cases, an active window of 0.5–1.8 s was compared to a control window spanning 7.0–8.3 s, relative to stimulus onset. Covariance matrices for beamformer reconstruction were calculated individually for the active and control windows, giving even amounts of data and thus ensuring equivalent accuracy (Brookes et al., 2008). The resulting pseudo-t-statistical images were used to derive the locations of the peak decrease in beta band oscillations in the left motor cortex and the peak increase in gamma oscillations in the visual cortex in each participant, which were used for further analyses (see Fig. 1e and f for a representative example). Note that since both peaks were identified from the active period contrasted with the baseline, the motor cortex (ERBD) peak was also used for analysis of the PMBR.

**Fig. 2:**
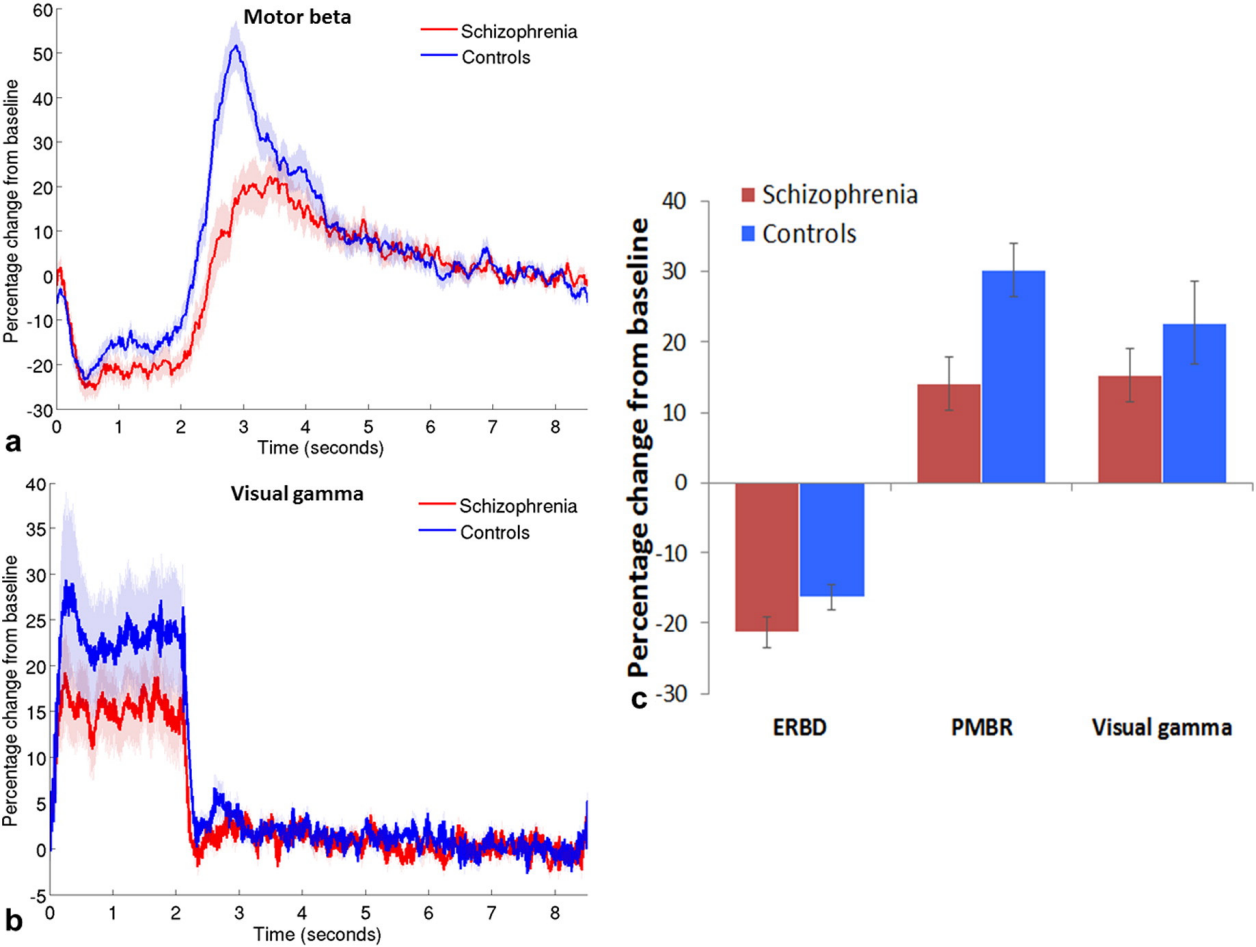
Beta and gamma band responses. Mean timecourse of beta band amplitude in motor cortex (a) and gamma band amplitude in visual cortex (b), measured as a percentage difference from baseline (7–8.3 s); shaded areas show standard error of the mean (SEM) across participants. c) Mean percentage signal change from baseline in motor cortex during event-related beta desynchronisation (ERBD; 0.5–1.8 s) and post-movement beta rebound (PMBR; 2.3–4.3 s); and in visual gamma oscillations during stimulation (0.5–1.8 s). Error bars represent SEM.

Virtual sensor timecourses were constructed for these peak locations also using a beamformer spatial filter. Beamforming was applied to data filtered into the 1–150 Hz band and the covariance matrix was generated using data spanning the entire experiment. The spatially filtered (virtual sensor) timecourses were sequentially filtered (temporally) into 23 overlapping frequency bands in the range of 1–100 Hz using a firls filter implemented in NUTMEG (http://nutmeg.berkeley.edu). For each band, the Hilbert envelope was calculated and averaged across task trials. A resting baseline signal was estimated as the mean Hilbert envelope within the 7.0–8.3 s window, relative to stimulus onset, and the percentage difference in signal from this baseline was calculated across the trial averaged timeseries for all frequency bands. These individual frequency bands were then concatenated to generate time frequency spectrograms, which were averaged across participants in the patient and control groups (Fig. 1). The percentage change from baseline for the beta desynchronisation was taken from the 0.5–1.8 s window, during which participants were moving, but which allowed time for participants to react to the stimulus onset. The beta rebound signal was taken from the 2.3–4.3 s window, based on the observed signal in a time frequency spectrogram averaged over all patients and controls. In both cases a 13–30 Hz frequency range was used. To test gamma band amplitude in the visual cortex, the percentage change from baseline was computed in the 30–70 Hz frequency window in the 0.5–1.8 s time window, in order to obtain only the sustained gamma response and not the initial gamma spike. These data are shown in Fig. 2. Pseudo t-statistical images for Fig. 1 were visualised using mri3dX (Singh, CUBRIC, Cardiff). Statistical analysis was carried out in SPSS (Armonk, NY: IBM Corp.) and MATLAB (The Mathworks Inc., Natick, MA).

## 3 Results

The task was well tolerated by all patients and controls. Across all 46 participants, two patients and two controls failed to show a well localised beta desynchronisation peak in the motor cortex and one patient did not show a clear gamma peak in visual cortex. These participants were excluded from analysis of these voxels, giving a total of 21 patients and controls contributing to analysis of motor beta, and 22 patients and 23 controls for visual gamma.

Fig. 1 shows time frequency spectrograms, averaged across task trials and participants, for patients and controls at the motor and visual locations of interest. Both groups exhibit the expected changes in oscillatory power in the beta and gamma bands throughout the trial: in the motor cortex, beta amplitude decreases during stimulation with a PMBR on movement cessation; and in the visual cortex, there is a decrease in alpha oscillatory amplitude and a concomitant increase in gamma amplitude during stimulation. As hypothesised, there were differences between patients and controls in features of these typical response profiles.

Figs. 2a and 2b show the timecourses of percentage signal change in oscillatory amplitude from baseline (7.0–8.3 s) for the beta band (13–30 Hz) in the motor cortex (a) and the gamma band (30–70 Hz) in the visual cortex (b) for patients (red) and controls (blue). The mean percentage changes during ERBD, PMBR and visual stimulation are shown in Fig. 2c for both groups. ERBD and visual gamma were measured in the 0.5–1.8 s window, the PMBR was measured from 2.3–4.3 s. Note that the largest difference between patients and controls is in the PMBR, which shows a 30% increase from baseline in controls, and only a 14% increase in patients.

**Fig. 3:**
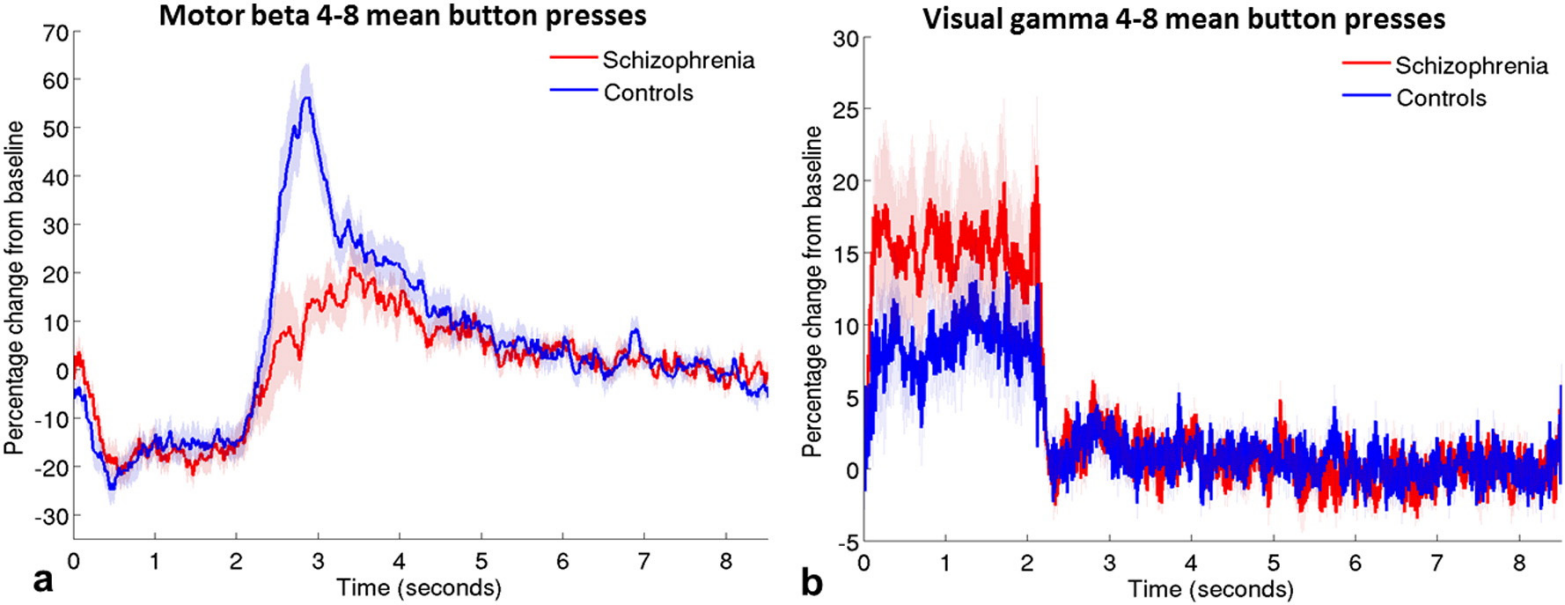
Timecourses for groups with equivalent numbers of button presses. Mean motor beta (a) and visual gamma (b) timecourses in groups of patients (red, *N* = 12) and controls (blue, *N* = 13) who made similar numbers of button presses (mean of 4–8 presses per trial). Shaded areas are SEM across participants.

Interestingly, behavioural data indicated that on average, patients made more button presses per trial (mean 6.83 presses, between subjects standard deviation (SD) 2.18, within subjects SD 1.03) than controls (mean 5.27 presses, between subjects SD 2.02, within subjects 0.60). This difference in these ‘button press counts’ was statistically significant (t(44) = −2.52; p = .016). However, the mean time of the last button press in each trial was similar for patients (1.92 s, SD 0.34) and controls (1.89 s, SD 0.19) (U = 33; p = .156). These results indicate that on average, patients with schizophrenia pressed the button more frequently in the allocated time period than controls but not for longer; therefore patients tended to press faster than controls. In order to investigate MEG data from groups with comparable behaviour, participants who pressed on average between four and eight times per trial were selected for initial analyses, based on the overlap of the distributions of button press counts from the two groups. This criterion gave groups of 12 controls and 13 patients. Responses from these subgroups are presented in Fig. 3. Note that the difference between groups in the PMBR remains, suggesting qualitatively that this effect is not simply accounted for by the different numbers of button presses in the two groups.

Statistical analysis of these behaviourally comparable data was conducted with a repeated measures analysis of variance (ANOVA), the results of which are shown in Table 3a. This analysis indicated that when beta amplitude was summed across both stages of the response, patients showed a slightly lower amplitude response than controls. However, the difference in beta amplitude between the groups was dependent on the stage (ERBD or PMBR) of the response, in that the two groups showed very similar ERBD but the PMBR was significantly reduced in patients. As expected, the PMBR was significantly greater than the ERBD in both groups. The difference in gamma amplitude between groups was analysed using a Mann–Whitney U-test, because these data were not normally distributed. There was no significant difference in gamma amplitude in the visual cortex between the two groups (U = 83; p = .810).

**Fig. 4:**
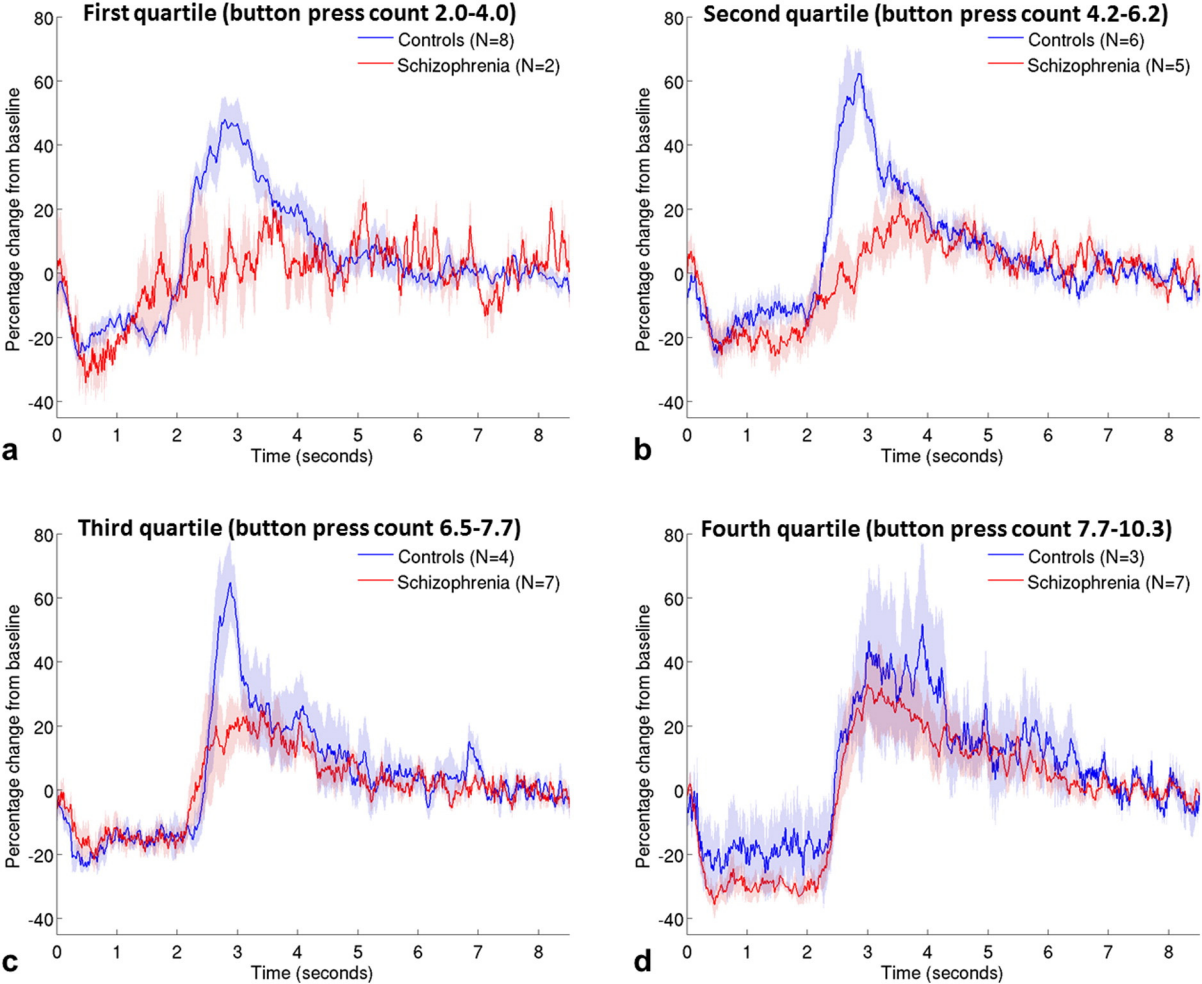
Effect of number of button presses. Mean beta timecourses for groups of patients (red) and controls (blue), defined by the quartiles of mean button press count across all volunteers, from lowest (a) to highest (d). Shaded areas represent SEM across all trials.

The difference between groups in the mean number of button presses made per trial (the button press count) warranted further investigation in relation to its impact on the MEG data from the full cohort of participants. An analysis of covariance was therefore conducted on the beta responses of all participants with button press count included as a covariate. The results of this analysis are presented in Table 3b. In this analysis, the overall difference between groups in the sum of beta amplitude across the two stages was not statistically significant; however, again there was a difference between the way in which the two groups responded at the two stages of the beta response (shown by the significant group by stage statistical interaction). Follow-up univariate ANCOVAs were conducted on each group separately and on each stage separately. They indicated that whilst the mean amplitudes of ERBD and PMBR were not significantly different between the groups, controls showed a significant increase from ERBD to PMBR (the peak-to-peak ‘beta difference’), irrespective of the number of button presses, whereas in patients, the beta difference was related to button press count. Individuals who pressed the button more often showed a greater increase from ERBD to PMBR (correlation between beta difference and button press count R^2^ = 0.23; p = .029). This effect can be observed in Fig. 4, which shows that for patients in the lowest quartile of mean button press counts, there is little change between the two beta stages, but as button press count increases, the beta difference in patients increases and the timecourse becomes more similar to controls. Controls show similar timecourses regardless of how many times they press the button (correlation of beta difference with button press count R^2^ = 0.02; p = .494). Fig. 4 again suggests that the PMBR is more affected by schizophrenia than ERBD; however there are insufficient numbers of participants to statistically test the group difference on the two stages across different button press counts.

Visual gamma was analysed using a univariate ANCOVA with mean button presses as a covariate. There was no significant difference between patients and controls' visual gamma amplitude (F(1,41) = .08; p = .780) and the button press count did not influence visual gamma (main effect of button presses: F(1,41) = 1.45; p = .236, and interaction between group and button presses: F(1,41) = .64; p = .428).

To investigate the influence of medication on the electrophysiological measures obtained, in a separate analysis of only the patient group, defined daily dose (DDD; WHOCCfDSM, 2012) of psychotropic medication was considered as an additional covariate in the contrast between beta stages. Taking DDD into consideration did not alter the results: patients with lower button press counts showed a smaller beta difference than those with higher button press counts, regardless of their dose of antipsychotic medication (significant beta stage × button press count interaction (F(1,17) = 5.04; p = .038); non-significant main effects of beta stage, button press count and DDD, and all other interactions between these variables (all F(1,17) < 1.89); p > .187). To assess the relationship between DDD and gamma, the correlation between 10,000 randomly paired gamma and DDD values was obtained, and the measured correlation was compared with the upper and lower 2.5% of values in the resulting distribution of correlations, to assess the probability of obtaining a correlation of the measured strength. DDD did not relate significantly to gamma amplitude in patients (R = −0.07; p = ns; 95% CI [−0.367 and 0.486]). These findings indicate that medication did not have a significant effect on motor beta or visual gamma oscillations.

**Fig. 5:**
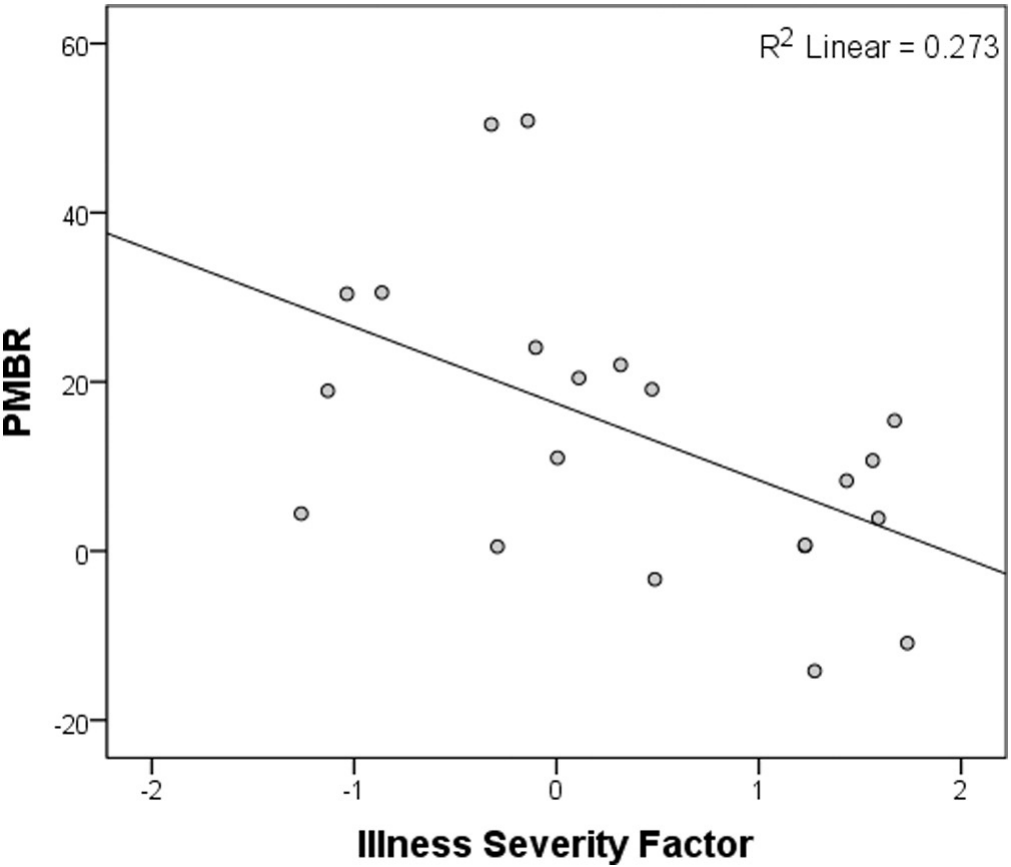
Correlation between PMBR and severity of persisting psychotic illness. The amplitude of the post-movement beta rebound showed a significant negative correlation with a measure of overall psychotic illness severity persisting during a stable phase of illness in the patient group.

Scores of overall severity of persisting psychotic illness exhibited a significant negative Pearson correlation with the PMBR in the patient group (R = −0.52; p = .015; Fig. 5), with no correlation between illness severity and ERBD or visual gamma (R = −.18 and .06 respectively; p = ns). The scores for the three core syndromes of schizophrenia loaded positively on the illness severity measure, whilst social, occupational and cognitive function loaded negatively on it (see Table 4), so those patients with higher severity scores had stronger core symptoms and lower levels of function. These were also the patients who showed the smallest beta rebound.

An additional interesting feature of the time–frequency spectrograms (Fig. 1) was the apparent difference in theta oscillations between groups. These were not part of our hypothesis, but were contrasted in non-planned post-hoc comparisons of the stimulation period (0.5–1.8 s). Patients showed significantly reduced theta in motor cortex (U = 78; p < .001), whilst the difference in visual cortex was not significant (t(43) = .62; p = .542).

In summary, the results indicate that in groups of participants matched for performance on a self-paced button press task, the amplitude of post-movement beta oscillations is reduced in patients with schizophrenia compared with controls. Beta reactivity, reflected in the change from the desynchronisation during movement to synchronisation following movement was reduced in patients who pressed the button less often. Visual gamma did not differ significantly between groups. Theta oscillations were reduced in patients' motor but not visual cortex.

## 4 Discussion

Deficits in sensorimotor function have been a well-established feature of schizophrenia since the earliest descriptions of the disease; however, despite their prevalence and impact, few studies have probed the neuronal mechanisms underlying these symptoms. In this study, we measured the electrophysiological signature of visual and motor processing in patients with schizophrenia and matched healthy control subjects using MEG. Our results show that the characteristic profiles of oscillatory responses to visual and motor stimulation are preserved in schizophrenia. However, significant differences in neuronal dynamics are observed in patients relative to controls. Specifically, the well-characterised temporal signature of beta oscillations in motor cortex during finger movement differs between the two groups: when matched for behaviour, patients showed a reduced PMBR, whilst their ERBD was relatively preserved. Interestingly, patients who pressed the button infrequently in our self-paced motor task showed significantly less of a difference between the ERBD and PMBR stages of the trial than those who pressed very rapidly. Patients who pressed rapidly showed beta timecourses that were similar to controls, in whom clear ERBD and PMBR responses were present regardless of their mean button press count. There was no significant difference in visual gamma oscillations between groups. Our results therefore indicate abnormalities in basic sensorimotor processing in patients with schizophrenia.

The differences in beta oscillatory response profiles between patients and controls provide a potential neuronal correlate of known motor disturbances in schizophrenia. There are various theories as to the roles that beta desynchronisation and rebound play in the generation and inhibition of movement. At rest, beta oscillations in motor cortex may control tonic contractions involved in maintenance of posture, whilst simultaneously inhibiting additional movements (Gilbertson et al., 2005). Decreases in beta synchrony during or preceding a movement may therefore reflect a switch to a state in which a greater range of movements can be made, since reduced synchrony allows greater flexibility to encode information in cellular assemblies (McIntosh et al., 2008). The increase in beta power following a movement has been suggested to reflect inhibition of motor activity: voluntary movements are slowed both during periods of beta oscillations as measured using electrocorticograms (Gilbertson et al., 2005), and when beta rhythms are entrained using transcranial alternating-current stimulation (Pogosyan et al., 2009). This post-movement inhibition may facilitate motor control by preventing repetition or generation of further movements and returning postural stability. Evidence for this theory comes from findings that the PMBR is almost absent in young children but increases through development (Gaetz et al., 2010); whilst in individuals with Parkinson's disease whose movements are limited and poorly controlled, both ERBD and PMBR are reduced in amplitude compared with controls (Heinrichs-Graham et al., 2014; Pollok et al., 2012). Beta oscillations may also reflect long-range communication between brain regions, whereas gamma oscillations reflect more local processing (Donner et al., 2011). In the context of these theories, the reduced reactivity of motor cortex in schizophrenia observed in this study may reflect maintenance of tonic contractions and reduced flexibility of responses during movements; reduced inhibitory control allowing efficient termination of the movement; and/or limited ability to switch between long-range and local communication between neurons.

Growing evidence suggests that both beta and gamma oscillations in the cortex depend upon a delicate balance between excitation and inhibition (Brunel et al., 2003; Kopell et al., 2000), governed largely but not exclusively by glutamate and gamma-aminobutyric acid (GABA), which are the principal excitatory and inhibitory neurotransmitters, respectively. There is a large amount of evidence for disruption to this balance in schizophrenia caused by GABAergic abnormalities (Lewis, 2014; Rowland et al., 2008), including reductions in levels of GAD, the enzyme necessary for synthesis of GABA from glutamate, and in the mRNA that codes for GAD and for the GABA transporter and receptors (Shin et al., 2011). There are also reductions in the volume of pyramidal neurons (Sweet et al., 2004) and in the number of parvalbumin expressing GABAergic inhibitory interneurons, which are involved in the generation of gamma oscillations (Lewis, 2014). Deletion of a gene coding for a receptor for neuregulin molecules, which are involved in regulating neuronal development in parvalbumin interneurons, causes reorganisation of cortical networks, increases oscillatory activity particularly in the gamma frequency and leads to schizophrenia-like symptoms including disruption of emotional and social behaviour and cognitive function in mice (Del Pino et al., 2013). Restoring normal levels of neuregulin reverses these symptoms (Marin et al., 2013). These animal studies strongly suggest a link between abnormalities in parvalbumin interneurons and schizophrenia.

In contrast with previous findings (Grutzner et al., 2013; Shin et al., 2011; Spencer et al., 2004; Sun et al., 2013), our study did not show significant reductions in gamma oscillations in the visual cortex of patients with schizophrenia, although the difference observed was in this direction. The link between GABAergic inhibition and beta oscillations has been assessed by an in vitro study showing generation of beta oscillations in neuronal assemblies of cortical layer V (Roopun et al., 2006), a magnetic resonance spectroscopy (MRS) study showing a positive correlation between GABA and PMBR power (Gaetz et al., 2011) and a pharmacological study showing that blocking GABA uptake increases ERBD and decreases PMBR amplitude (Muthukumaraswamy et al., 2013). Impaired GABAergic neurotransmission in schizophrenia may therefore contribute to the decrease in amplitude of beta oscillations observed in patients in our study.

In patients, the difference between beta desynchronisation and rebound was greater and more similar to controls when they pressed the button at a faster rate (Fig. 4). PMBR in response to median nerve stimulation is reduced by ERBD generated during simultaneous movement, and this effect is greater as the amount of motor activity increases (e.g. passive stretch compared with exploratory finger movement (Salenius et al., 1997), or imagined compared with performed movement (Schnitzler et al., 1997)), but direct measurement of the effects of movement complexity or frequency on the movement-related beta response have not to our knowledge been reported. The relative poverty of motor responses in some of the patients with schizophrenia in this study may arise from the observed abnormalities in beta reactivity. Such an effect could be due to impaired communication between motor cortex and higher order cortical regions, and/or to altered neurotransmission; however, these suggestions are speculative and would require further study using measures of functional connectivity and transmitter concentration or cycling rates. On average, patients tended to exhibit a higher button press count compared with controls. It is not clear why this was the case; it may represent a form of coping strategy, given that with higher button press counts, the beta timecourse became more similar to controls. This behaviour somewhat masked abnormalities in beta oscillations that would likely have been more striking had only one button press been required.

Use of a task with self-paced movements and subsequent analysis of data taking into consideration the button press count has revealed interesting differences in the beta oscillatory profiles between the two groups that would not otherwise have been evident. Another significant advantage of the paradigm presented here is that it is short, simple and is suitable for individuals of all cognitive abilities. The ability to measure differences between patients with schizophrenia and controls in such a basic paradigm is promising for eventual translation of similar approaches into clinical practice. This MEG approach, which permits investigation of neuronal activity within brain networks of patients and healthy volunteers, also reduces the need to conduct similar studies in animal models of mental health disorders.

It is important to consider the effect of medication on the electrophysiological measures obtained in this study. The patients were primarily taking antipsychotics, which inhibit dopaminergic function (Castle et al., 2013) and some were taking antidepressants, which enhance serotonergic or noradrenergic mechanisms (Feighner, 1999). Because dopamine has an inhibitory effect on cortical activity (Ciccone, 2015), antipsychotics tend to shift the balance of activity towards cortical excitation. There is evidence from preclinical studies that chronic administration of antipsychotics can reduce steady state oscillatory power in the gamma frequency (Anderson et al., 2014), whilst non-invasive neuroimaging has shown enhanced steady state delta and theta but reduced alpha and beta amplitude after administration of antipsychotics in healthy volunteers (Galderisi et al., 1996; Hubl et al., 2001). The observation that taking into account patients' daily dose of psychotropic medication did not alter the results, together with evidence from other studies indicating that oscillatory abnormalities are present in unmedicated patients with schizophrenia (Boutros et al., 2008; Gallinat et al., 2004; Sun et al., 2013) and in first degree relatives (Hong et al., 2012) suggests that the abnormalities observed in our study are unlikely to be attributable to medication. Nevertheless, further investigation into the impact of medication on the responses measured in this study is warranted.

The PMBR was inversely correlated with a score of overall severity of psychotic illness assessed during a stable phase of the illness. The scores for the three core syndromes of schizophrenia loaded positively on the illness severity measure, whilst social, occupational and cognitive function loaded negatively on it (Table 4). The magnitude of the DSST loading is lower than for the other items, but DSST score was retained because the set of items were selected a priori as a measure of illness severity (Palaniyappan et al., 2013a) and a meta-analysis of cognitive impairments in schizophrenia has shown that the DSST quantifies an inefficiency of information processing that is an important feature of schizophrenia (Dickinson et al., 2007).

Similar to previous studies (e.g. Liddle, 1987b), the three syndrome scores showed low mutual correlations (0.033–0.33; p < .05), but all three loaded heavily on a single factor, along with the SOFAS and DSST scores, indicating that they are associated with a latent variable likely to reflect severity of illness. Scores on this composite measure of overall current severity of psychotic illness exhibited a significant negative Pearson correlation with the PMBR in the patient group (R = −0.52; p = .015; Fig. 5), with no correlation between illness severity and ERBD or visual gamma (R = −.18 and .06 respectively; p = ns). Therefore, those patients with higher severity scores had stronger core symptoms and lower levels of function and these were also the patients who showed the smallest beta rebound.

In conclusion, abnormalities in perceptual processing and psychomotor performance are key aspects of the pathophysiology that influences functional outcome in schizophrenia (Boden et al., 2014; Javitt, 2009). Electrophysiological biomarkers for these deficits can be identified using non-invasive neuroimaging techniques (Javitt et al., 2008) and they can be modified using targeted training (Adcock et al., 2009). We have used a simple visuomotor task and MEG imaging to show abnormalities in the dynamics of beta oscillations in motor cortex, giving direct electrophysiological evidence to support theories of impaired cortical inhibition in schizophrenia. Consistent with our current understanding of GABA–glutamate dysfunction in this illness, our findings raise the possibility of targeting the visuomotor system for behavioural training and pharmacological treatments in patients with schizophrenia.

## Figures and Tables

**Table 1:** National Statistics Socio-economic Classification (NS-SEC) scores.

	NS-SEC score	1	2	3	4	5	Mean	SD
Number of participants	Schizophrenia	13	1	4	1	4	2.2	1.6
	Controls	11	2	5	3	2	2.3	1.4

**Table 2:** Details of patients' pharmacological treatment including the drug, its dose and the total defined daily dose (DDD) of psychotropic medication for each participant. Doses are per day unless given as a depot, in which case the frequency is specified.

Participant	Drug (dose)	Total DDD
1	Risperidone (25 mg/1–2 weeks); Citalopram (20 mg)	1.66
2	Risperidone (2 mg)	0.4
3	Diazepam (2 mg); Mirtazapine (45 mg); Aripiprazole (20 mg); Zopiclone (7.5 mg)	4
4	Amisulpride (200 mg); Clozapine (275 mg)	1.42
5	Olanzapine (15 mg)	1.5
6	Olanzapine (25 mg); Sertraline (50 mg)	3.5
7	Lofepramine (70 mg)	0.67
8	Paliperidone (100 mg/month); Risperidone (3 mg)	1.15
9	Mirtazapine (45 mg); Aripiprazole (20 mg); Pregabalin (150 mg)	3.3
10	Clozapine (400 mg)	1.3
11	Olanzapine (5 mg)	0.5
12	Aripiprazole (20 mg)	1.33
13	Quetiapine (200 mg)	0.5
14	Clozapine (350 mg), Sulpiride (200 mg)	1.42
15	Aripiprazole (5 mg)	0.33
16	Risperidone (2 mg), Sertraline (100 mg)	2.4
17	Clozapine (400 mg), Sulpiride (400 mg)	1.8
18	Sertraline (200 mg), Lithium (1 g), Clozapine (200 mg)	5.77
19	Zuclopenthixol (20 mg)	2.86
20	Olanzapine (20 mg)	2
21	Quetiapine (200 mg), Procyclidine (5 mg), Venlafaxine (75 mg), Modecate (25 mg/2 weeks)	2.45
22	Clozapine (300 mg)	1
23	Aripiprazole (10 mg)	0.67
	Mean	1.82
	SD	1.34

**Table 3:** Results of a repeated measures ANOVA on beta amplitude in the subgroup with comparable behavioural responses (a), with the between subjects factor of group (patients and controls) and within subjects factor of beta stage (ERBD and PMBR). The same contrasts but with the covariate of mean button press count are presented in the ANCOVA results, which includes data from all participants (b). * Denotes significance at 5% level (*p* < 0.05); ** denotes significance at 1% level (*p* < 0.01).

(a)		
ANOVA on beta amplitude		
Main effect: group	ERBD Controls vs patients	PMBR Controls vs patients
F(1,23) = 4.95; p = .036*	t(23) = .34; p=.740	t(23) = 2.77; p = .011*


Follow-up contrasts (t-tests)			
Controls ERBD vs PMBR	Patients ERBD vs PMBR	Main effect: beta stage	Interaction: group by stage
t(11) = −9.56; p < .001**	t(12) = −7.62; p < .001**	F(1,23) = 151.65; p < .001**	F(1,23) = 7.01; p = .014*


(b)					
ANCOVA on beta amplitude, covariate of mean button press count					
Main effect: group	Main effect: beta stage	Main effect: button press	Interaction: group by stage	Interaction: group by button press	Interaction: group by stage by button press
F(1,38) = 0.83; p = .368	F(1,38) = 10.61; p = .002*	F(1,38) = 0.17; p = .679	F(1,38) = 6.29; p = .017*	F(1,38) = 0.01; p = .937	F(1,38) = 3.19; p = .082

Follow-up contrasts (univariate ANCOVAs, covariate of mean button press count)					

ERBD			PMBR		

Main effect: group	Main effect: button press	Interaction: group by button press	Main effect: group	Main effect: button press	Interaction: group by button press

F(1,38) = 1.40; p = .244	F(1,38) = 0.63; p = .432	F(1,38) = 2.96; p = .094	F(1,38) = 3.21; p = .081	F(1,38) = 0.91; p = .346	F(1,38) = 0.66; p = .422

Controls			Patients		

Main effect: beta stage	Main effect: button press	Interaction: beta stage by button press	Main effect: beta stage	Main effect: button press	Interaction: beta stage by button press

F(1,19) = 17.72; p < .001**	F(1,19) = 0.13; p = .725	F(1,19) = 0.06; p = .815	F(1,19) = 0.28; p = .606	F(1,19) = 0.06; p = .816	F(1,19) = 6.11; p = .023*

**Table 4:** Loadings on the first factor derived from factor analysis of clinical features hypothesised to reflect current severity of illness: reality distortion, psychomotor poverty and disorganisation syndromes from the Signs and Symptoms of Psychotic Illness (SSPI) scale (Liddle, 2002); and scores from the Digit Symbol Substitution Test (DSST; Wechsler, 1940) and the Social and Occupational Function Assessment Scale (SOFAS; APA, 1994).

Illness severity measure	Loading on severity of persisting illness factor
Reality distortion	0.72
Psychomotor poverty	0.61
Disorganisation	0.58
DSST	−0.37
SOFAS	−0.67
